# Development of Chemically Defined Media Reveals Citrate as Preferred Carbon Source for *Liberibacter* Growth

**DOI:** 10.3389/fmicb.2018.00668

**Published:** 2018-04-05

**Authors:** Maritsa Cruz-Munoz, Joseph R. Petrone, Alexa R. Cohn, Alam Munoz-Beristain, Nabil Killiny, Jennifer C. Drew, Eric W. Triplett

**Affiliations:** ^1^Microbiology and Cell Science Department, Institute of Food and Agricultural Sciences, University of Florida, Gainesville, FL, United States; ^2^Citrus Research and Education Center, Institute of Food and Agricultural Sciences, University of Florida, Gainesville, FL, United States

**Keywords:** chemically defined medium, metabolism, biochemical composition, culturing

## Abstract

*Liberibacter crescens* is the closest cultured relative of four important uncultured crop pathogens. *Candidatus* L. asiaticus, L. americanus, and L. africanus are causal agents of citrus greening disease, otherwise known as huanglongling (HLB). *Candidatus* L. solanacearum is responsible for potato Zebra chip disease. Cultures of *L. crescens* grow slowly on BM-7 complex medium, while attempts to culture the *Ca.* Liberibacter pathogens in BM-7 have failed. Developing a defined medium for the growth of *L. crescens* will be useful in the study of *Liberibacter* metabolism and will improve the prospects for culturing the *Ca*. Liberibacter pathogens. Here, M15 medium is presented and described as the first chemically defined medium for the growth of *L. crescens* cultures that approaches the growth rates obtained with BM-7. The development of M15 was a four step process including: (1) the identification of Hi-Graces Insect medium (Hi-GI) as an essential, yet undefined component in BM-7, for the growth of *L. crescens*, (2) metabolomic reconstruction of Hi-GI to create a defined medium for the growth of *L. crescens* cultures, and (3) the discovery of citrate as the preferred carbon and energy source for *L. crescens* growth. The composition of M15 medium includes inorganic salts as in the Hi-GI formula, amino acids derived from the metabolomic analyses of Hi-GI, and a 10-fold increase in vitamins compared to the Hi-GI formula, with exception choline chloride, which was increased 5000-fold in M15. Since genome comparisons of *L. crescens* and the *Ca*. Liberibacter pathogens show that they are very similar metabolically. Thus, these results imply citrate and other TCA cycle intermediates are main energy sources for these pathogens in their insect and plant hosts. Thus, strategies to reduce citrate levels in the habitats of these pathogens may be effective in reducing *Ca.* Liberibacter pathogen populations thereby reducing symptoms in the plant host.

## Introduction

The uncultured *Candidatus* Liberibacter asiaticus and *Ca.* L. solanacearum are Gram-negative, phloem-limited, insect-vectored plant pathogens in the α-proteobacteria that are responsible for the economically devastating citrus greening and potato Zebra chip (ZC) diseases around the world (Wang et al., 2017). The fastidious nature of these pathogens has limited our ability to grow them in axenic culture, which in turn has limited the studies on disease etiology, disease mechanisms, disease control methods, as well as hindering the fulfillment of Koch’s postulates (Hall et al., 2013; Cooper et al., 2014). Reduced genomes with limited metabolic potential, restricted ecological niches, and slow-growing capabilities are hallmarks of the obligate intracellular lifestyle of these pathogens (Zarecki et al., 2014). Only one species from the genus *Liberibacter, L. crescens* can be grown in culture in a complex medium, BM-7, which contains fetal bovine serum (FBS) and the enriched insect TNM-FH (Fagen et al., 2014b). To date, there are no reproducible reports of the culture growth of any of the *Ca*. Liberibacter pathogens in BM-7 or any other medium.

Being the closest cultured relative to the *Ca*. Liberibacter pathogens, *L. crescens* is the model microorganism to study the lifestyle, virulence, antimicrobial susceptibility, and culturability of these bacteria. Genome comparisons between *L. crescens* and non-culturable *Liberibacter* pathogens suggest that these strains are very similar metabolically (Fagen et al., 2014a; Wulff et al., 2014). An essential gene set of *L. crescens* was prepared by transposon mutagenesis to identify those essential genes that are absent in the *Ca*. Liberibacter pathogens (Lai et al., 2016). Those *L. crescens* essential genes that are absent in the pathogens may be conferring the ability of *L. crescens* to grow on BM-7. The lack of these genes suggests that the uncultured pathogens acquire aromatic amino acids, vitamins, histidine, cysteine, lipopolysaccharides, and fatty acids from their host environments (Lai et al., 2016). The *L. crescens* strain grows slowly on BM-7, taking 8 days to pass through the different phases of growth, with a doubling time of 36.7 h at 28°C (Fagen et al., 2014b). The *Liberibacter* model species was also used to evaluate the function of several *Ca.* L. asiaticus proteins important for growth and as biochemical targets (Fleites et al., 2014; Pagliai et al., 2014; Jain et al., 2015; Loto et al., 2017). A defined medium for *L. crescens* will allow investigators to test specific media components to improve the culture growth of *L. crescens*, which is a valuable step toward culturing the *Ca*. Liberibacter pathogens.

Recent studies suggest that *Liberibacter* pathogens obtain energy by exploiting TCA cycle intermediates, and by scavenging ATP from their hosts (Wang and Trivedi, 2013; Jain et al., 2017; Killiny et al., 2017b). Oxidation of sugars through glycolysis is probably avoided because *Ca*. Liberibacter pathogens lack the glycolytic enzyme phosphoglucose isomerase and the glyoxylase system (Fagen et al., 2014b; Jain et al., 2017). The first catalyzes the interconversion of glucose-6-phosphate and fructose-6-phosphate, and the latter contributes to detoxify excess of methylglyoxal generated through glycolysis (Jain et al., 2017). Additionally, the overexpression of citrate related enzymes of *Ca.* L. asiaticus and the abundant citrate present in HLB-infected psyllid hemolymph suggest this pathogen utilizes citrate for energy production (Killiny et al., 2017b). The high concentrations of citrate and other carboxylates in the citrus phloem also support the notion that citrus phloem is an excellent habitat for *Ca.* L. asiaticus (Killiny, 2017).

Here a series of steps is described that led to the development of M15 medium, the first chemically defined medium for the growth of *L. crescens.* M15 medium uses citrate as the main carbon and energy source. The development of this defined medium allowed testing of a variety of carbon sources for improved growth of *L. crescens* cultures which has led to mechanism by which a citrus nutritional strategy might reduce severity of citrus greening disease.

## Materials and Methods

### Reagents, Bacterial Strains, and Culture Conditions

TNM-FH insect medium was purchased through HyClone (Logan, UT, United States). FBS was through VWR Life Science Seradigm (Radnor, PA, United States). Grace’s Insect Media was purchased through HiMedia (Mumbai, India), Gibco (Thermo Fisher Scientific, Waltham, MA, United States), and Sigma-Aldrich (St. Louis, MO, United States). All other reagents were obtained through Sigma-Aldrich (St. Louis, MO, United States), unless specified. *L. crescens* strain BT-1 was used in all experiments, and growth was monitored at 28°C and 150 rpm. BM-7 complex medium was prepared as follows: 2 g of α-ketoglutarate, 10 g of ACES buffer, and 3.75 g of KOH were mixed in 550 mL of deionized water with a final pH of 6.9. After autoclaving and cooling to 50°C, 150 mL of sterile FBS and 300 mL of sterile TNM-FH are added to complete 1 L of the medium (Fagen et al., 2014a).

### Metabolomics Analysis in Hi Grace’s Insect Medium (Hi-GI)

Metabolomics analysis of the three commercial sources of Hi-GI media was performed according to previously described protocols (Killiny, 2017; Killiny et al., 2017a). Samples were derivatized using Trimethylsilyl (TMS) and Methyl chloroformate (MCF) prior gas chromatography–mass spectrometry (GC–MS) to cover up for all the compounds able to detect through this technique.

### Trimethylsilyl (TMS) Derivatization

An aliquot of 10 μL of filtered-sterilized Hi-GI medium was mixed with 250 μg/mL final concentration of dodecanoic acid (as internal standard). The mixture was subsequently transferred to a 2 mL GC vials with 200 μL fused inserts (National Scientific, Rockwood, TN, United States) prior freeze-drying under nitrogen stream. Aliquots of 10 μL of 0.1% (w/v) phenylthiourea with decanoic acid were also dried and used as blank samples. Subsequently, samples were mixed with 5 μL of methoxamine hydrochloride in pyridine reagent and incubated for 16 h. Finally, samples were mixed with 10 μL of *N*-methyl-*N*-trimethylsilyl-trifluoroacetamide and incubated for 2 h at 25°C, prior injection. 0.3 μL were injected into GC injector port at 250°C for separation. Due to high contents of sugars in the medium, MCF was also performed cover up for all amino acids.

### Methyl Chloroformate (MCF) Derivatization

An aliquot of 30 μL of filtered-sterilized Hi-GI medium and authentic standards were mixed with 180 μL of 1N sodium hydroxide in 1 mL salinized conical insert. Subsequently, samples were mixed with 167 μL of methanol and 34 μL of pyridine. Then, 20 μL of MCF were added twice followed by 30 s of vigorous agitation. Chloroform (100 μL) were added to stop the reaction with vigorous mixing for 10 s. Sodium bicarbonate (100 μL of 50 mM) were also added and mixed for 10 s. The mixture was then placed into a new insert vial and few crystal of sodium sulfate were added, prior to injection of 1 μL into the GC injector port at 250°C for analysis.

### GC–MS Conditions

Samples were processed through a PerkinElmer Autosystem XL GC–MS with Turbomass software in electron impact mode at 70 eV, with full scans of 40–600 Da and a ZB-5MS capillary column of 30 m × 0.25 mm × 0.25 μm film thickness (Phenomenex, Torrance, CA, United States). Ultra-pure hydrogen gas was used as a carrier at 0.9 mL min^-1^ flow rate. The GC oven conditions were: 70°C for 1 min, increased to 220°C for 1 min, and finally to 300°C, with increments of 10°C min^-1^ in both intervals. GC interface was set at 180°C while the MS detector at 150°C.

### Compound Identification and Quantification

Compounds were first identified using NIST 2011 and Wiley (9th edition) mass spectral libraries to compare mass spectra and retention times. Confirmation was also performed with authentic reference standards. Standard curves of TMS or MCF derivatized standards at dilutions of 2000, 1000, 500, 250, and 125 mg/l were used for compound quantification. Normalization to the mean of area of the internal standard compensated for any potential variations within all analytical procedures.

### Defined Media Preparation and *L. crescens* Growth Assays

Trimethylsilyl and MCF derivatized compounds were considered for the preparation of defined media. Cofactors and vitamin concentrations were increased to 10-fold compared to those of Hi-GI, except for choline chloride, which was increased 5000-fold. Inorganic salts were kept at the same concentration listed in Hi-GI since these are not detectable through GC–MS. All compounds were soluble when mixed together, except for tyrosine, which was dissolved in 1 M HCl before addition into the media. The medium was adjusted slowly to pH 5.92 by titration with 5 M KOH prior to sterilization. For all experiments, glycerol stocks of *L. crescens* in Hi-GI were used to inoculate media for growth to stationary phase (OD_600_∼0.8). Growth was monitored using optical density (OD_600_) twice a day for up to 12 days using the Sinergy BioTek Multi-mode microplate reader (BioTek Instruments, Inc.). Citrate concentrations tested in M14 were a range of those found in different citrus phloem varieties (200–6400 mg/L), as well as the concentration found in the *D. citri* hemolymph (9.6 mg/L). All culture treatments were done in triplicate. Recipes for M13 and M14 media are provided in Supplementary Table S1. The recipe for M15 is provided in **Table 1**.

**Table 1:** The constituents of M15 medium, a defined medium for the growth of *L. crescens*.

Components	M15
**Inorganic salts**	
Calcium chloride dihydrate	1320
Magnesium chloride anhydrous	1068.2
Magnesium sulfate anhydrous	1356.7
Potassium chloride	2240
Sodium phosphate monobasic monohydrate	1007
**Amino acids**	
β-Alanine	447.25
L-Alanine	447.25
L-Arginine-HCl	1777
L-Asparagine monohydrate	1075.45
L-Aspartic acid	818.6
L-Cystine-2HCl	56.38
L-Glutamic acid	1502.2
L-Glutamine	358.04
Glycine	859.512
L-Histidine hydrochloride monohydrate	2366.11
L-Isoleucine	687.36
L-Leucine	592.89
L-Lysine-HCl	1464.85
L-Methionine	678.9
L-Phenylalanine	789.62
L-Proline	940.61
DL-Serine	944.76
L-Threonine	459.8
L-Tryptophan	373.73
L-Tyrosine disodium salt	391.37
L-Valine	644.31
Betaine	0.31
Ornithine	229.96
Methionine sulfoxide	18.2
**Vitamins + Others**	
L-Biotin	0.1
Choline chloride	1000
Folic acid	0.2
Myo-inositol	0.2
Niacin	0.2
D-Calcium pantothenate	0.2
Para-aminobenzoic acid (PABA)	0.2
Pyrodoxine-HCl	0.2
Riboflavin	0.2
Thiamine-HCl	0.2
**Organic acids**	
Citric acid	2500
**pH**	5.92

*Concentrations are given in mg/L.*

## Results

The steps that led to the development of the first defined medium for *Liberibacter* and the discovery of citrate as the preferred carbon and energy course are reported below.

### Step 1: Hi-Grace’s Insect Medium (Hi-GI) Is Essential for *L. crescens* Growth

**FIGURE 1:**
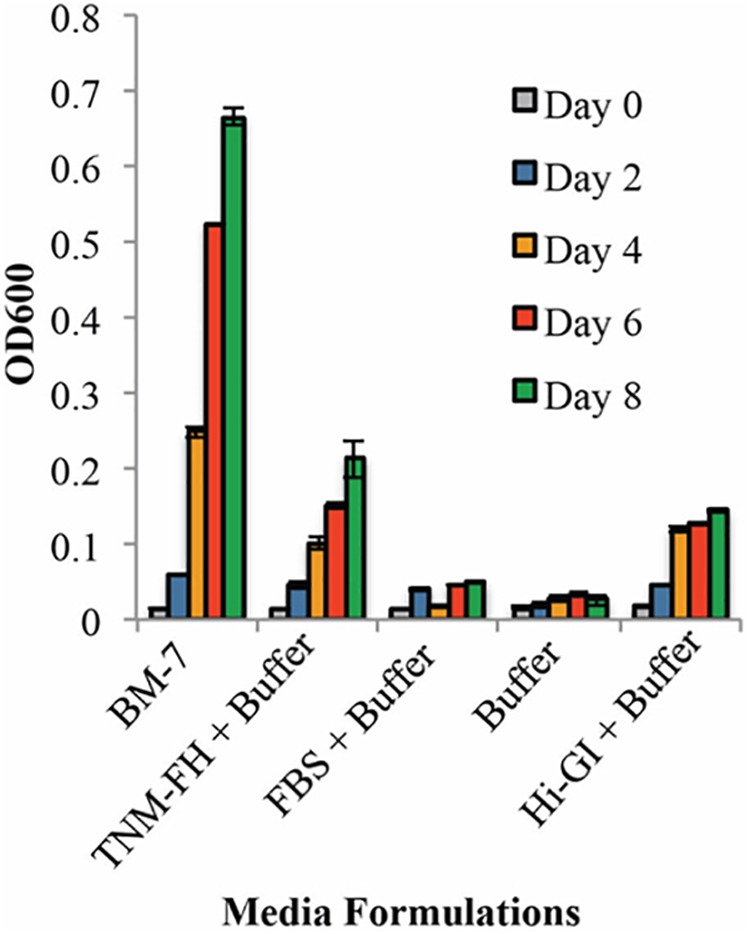
The growth of *Liberibacter crescens* in BM-7 components. Cells were grown in Hi-GI and washed prior inoculation. Growth was monitored measuring optical density (OD_600_) every 2 days for 8 days. The media containing TNM-FH and Hi-GI in the buffer system permit better growth of *L. crescens* compared to media with FBS in buffer or buffer alone. Growth in BM-7 was used as control.

Each component of the BM-7 complex medium was tested for its ability to sustain *L. crescens* culture growth. BM-7 contains a buffer (ACES, KOH, and α-ketoglutarate), an enriched insect TNM-FH medium, and FBS (Fagen et al., 2014b). In turn, TNM-FH is composed of a chemically defined Grace’s Insect Medium, lactalbumin hydrolysate (LAH), and yeastolate (YT) (Hink, 1991). When *L. crescens* BT-1 was grown in BM-7 with different sources of Grace’s insect medium, TNM-FH, Hi Grace’s Insect medium (Hi-GI), or Hi-media lab (Lab GI). BT-1 grew similarly in all three media in the buffered system [*F*(1,0.000315 = 0.29), *p* = 0.59, **Figure 1**]. BT-1 did not grow in buffered FBS or in buffer alone showing that some ingredients in Grace’s medium are required for growth (**Figure 1**). Buffered TNM-FH or Hi-GI sustained growth of *L. crescens* growth in the absence of FBS. As FBS was not required for growth, it could be omitted to create a defined medium. Since Hi-GI and TNM-FH can each support growth, TNM-FH was not included in future media because TNM-FH it is not defined. Thus, Hi-GI with buffering became the base defined medium for further improvement.

**FIGURE 2:**
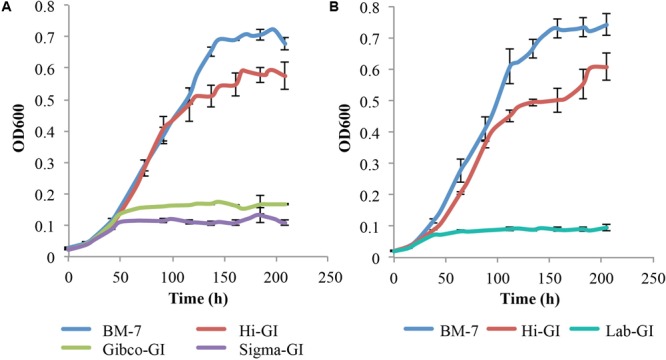
*Liberibacter crescens* growth in different Grace’s Insect media. Cells were grown in Hi-GI and washed prior inoculation. Growth was monitored measuring optical density (OD_600_) twice a day for ∼9 days. *L. crescens* grew significantly better in Hi-GI compared to Gibco-GI, Sigma-GI and Lab-GI media, and similar to BM-7 up to 116 h. **(A)** *L. crescens* growth in BM-7, Hi-GI (Hi-Media Labs), Gibco-GI (Gibco Technologies), and Sigma-GI (Sigma-Aldrich). **(B)** *L. crescens* growth in Grace’s Insect Medium prepared in the lab (Lab-GI) based on Hi-GI formula.

Since Grace’s insect media is chemically defined, the ability of *L. crescens* to grow in the three commercial sources of Grace’s insect media was tested (**Figure 2A**). The growth of *L. crescens* differed among the three insect media even though they are advertised as being of the same composition. To solubilize the Hi-GI media the pH had to be adjusted to 5.92. In these experiments, the Grace’s medium was added at a three-times higher concentration than was done for the cultures in **Figure 1**. Surprisingly, *L. crescens* only showed robust growth in Hi-GI, reaching an optical density similar to that in BM7 for the first 116 h (**Figure 2A**) [*F*(1,0.00286 = 1.861), *p* = 0.18]. After 116 h, *L. crescens* grew slightly faster in BM-7 while entering into a slow stationary phase in Hi-GI up to 209 h [*F*(1,0.2261 = 9.24), *p* = 0.0041, Figure2A]. The strain grew poorly in Gibco and Sigma Graces insect media (**Figure 2A**). The culture of the strain also failed to grow on a lab-prepared version of Grace’s insect medium prepared in the lab using the recipe for Grace’s insect media (Lab-GI, **Figure 2B**). The two growth curves in **Figure 2** differ slightly as they were done at different times. They show the slight variation that can occur between experiments. Hence, the ingredients in the source of Grace’s medium were determined to suggest how a better defined medium might be made.

### Step 2: Composition of Hi Grace’s Insect Medium (Hi-GI) Leads to the Design of a Defined Medium for *L. crescens*

To determine why the Hi-GI version of Grace’s insect medium performed so much better than the other sources for supporting the growth of *L. crescens* cultures, the metabolic constituents of each medium were determined by GC–MS. This quantitative metabolomics analysis of Hi-GI identified TMS and MCF derivatizations identified 23 and 22 commercially available compounds, respectively (Supplementary Tables S2–S4). TMS derivatization is the most commonly used silylation method for detection of sugars and their derivatives while MCF derivatization represents an alkylation method for detection of polyfunctional amines and organic acids using GC–MS (Villas-Boas et al., 2011). The combination of these two methods improved the detection and analysis of most metabolites in Hi-GI medium through GC–MS.

Based on the metabolomic analysis, the defined medium’s components, excluding the vitamins), were altered accordingly to make the M15 medium (**Table 1**). To complete the M15 recipe, all vitamin concentrations were increased 10-fold except for choline chloride, which was increased 5000-fold. These additions were based on empirical testing of *L. crescens* growth.

**FIGURE 3:**
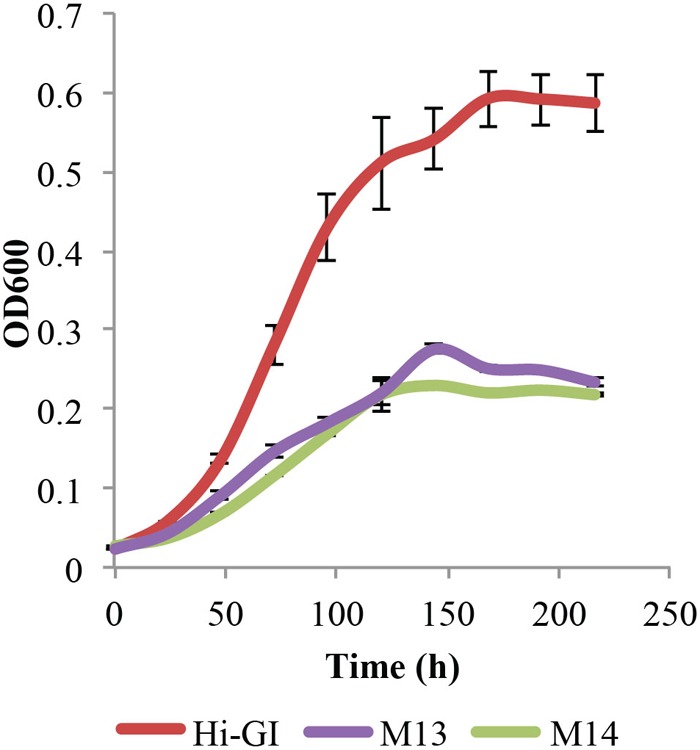
*Liberibacter crescens* growth in Hi-GI, M13, and M14 defined media. Cells were grown in Hi-GI and washed prior inoculation. Growth was monitored measuring optical density (OD_600_) twice a day for ∼9 days. *L. crescens* grew similar in M13 and M14, but significantly less than Hi-GI. M14 medium contains all of the components of M13 except glucose, fructose, turanose, maltose, fumarate, α-ketoglutarate, malate, maleate, and succinate.

However, *L. crescens* cultures grew for only two transfers in M13 defined medium, requiring further changes to the medium. As a result, various sources of carbohydrate were tested for their effect on *L. crescens* growth. Thus, a basal medium, M14, was created in which all sugars and organic acids were removed from M13 including glucose, fructose, turanose, maltose, fumarate, α-ketoglutarate, malate, maleate, and succinate. Cultures of *L. crescens* grew poorly in M13 and M14 (Tukey HSD, *p* = 0.744, **Figure 3**), suggesting that the medium needs to be supplemented with other compounds. The growth of *L. crescens* in M13 and M14 was significantly lower compared to Hi-GI (Tukey HSD, *p* = 0.0000, **Figure 3**). The constituents of M13 and M14 are listed in Supplementary Table S1.

### Step 3: Optimization of Citrate Levels for *L. crescens* Growth

**FIGURE 4:**
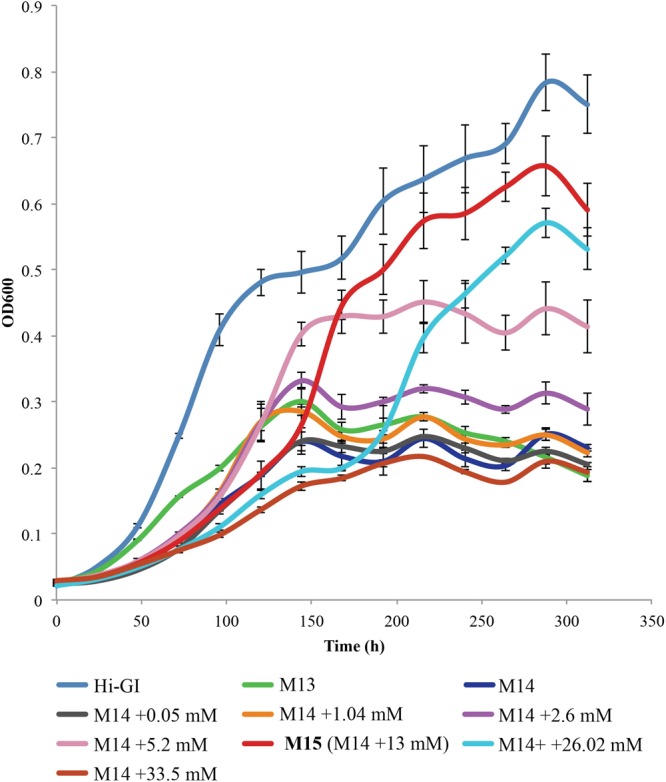
*Liberibacter crescens* growth tested in M14 medium with a range of citrate concentrations found in citrus phloem (200–6400 mg/L). Cells were grown in Hi-GI, and washed prior inoculation. Culture growth was monitored by measuring optical density (OD_600_) twice a day for ∼11 days.

A metabolic analysis of phloem in 14 citrus varieties showed that two organic acids, malate and citrate, were present in high concentrations (Killiny, 2017). Citrate was also the organic acid found in the highest concentration in the CLas-infected psyllid (Killiny et al., 2017a). Thus, the optimal level of citrate required for the culture growth of *L. crescens* was determined. Cells were cultured at various levels of citrate from 0 to 31.2 mM within M14 medium. Of the levels of citrate tested, the concentration of 13 mM was optimal for the growth of *L. crescens* cultures (**Figure 4**). The M14 medium containing 13 mM citrate is referred to as M15 medium (**Table 1**). In this medium, *L. crescens* grew slowly up to 134 h, after which rapid growth was achieved up to 285 h when the OD_600_ reached ∼0.65, very similar to growth on Hi-GI media (**Figure 4**). Very low (50 μM) or high levels (2.6 mM) of citrate resulted in much lower growth compared to the M14 medium without citrate (Tukey HSD, *p* = 1, *p* = 0.98, *p* = 0.47, respectively). With 5.2 mM citrate, *L. crescens* culture growth was not significantly different compared to M15 (Tukey HSD, *p* = 0.224) up to 158 h (OD_600_∼0.4) after which growth in M15 increased significantly over the lower level of citrate (Tukey HSD, *p* = 0.000, **Figure 4**). Levels of citrate above 13 mM reduced growth (Tukey HSD, *p* = 0.025, *p* = 0.0000, **Figure 4**). With 13 mM citrate added as a carbohydrate source, the M14 medium is referred to as M15.

### Step 4: Investigation of Carbohydrate Sources Preferred by *L. crescens* Using a Defined Medium

**FIGURE 5:**
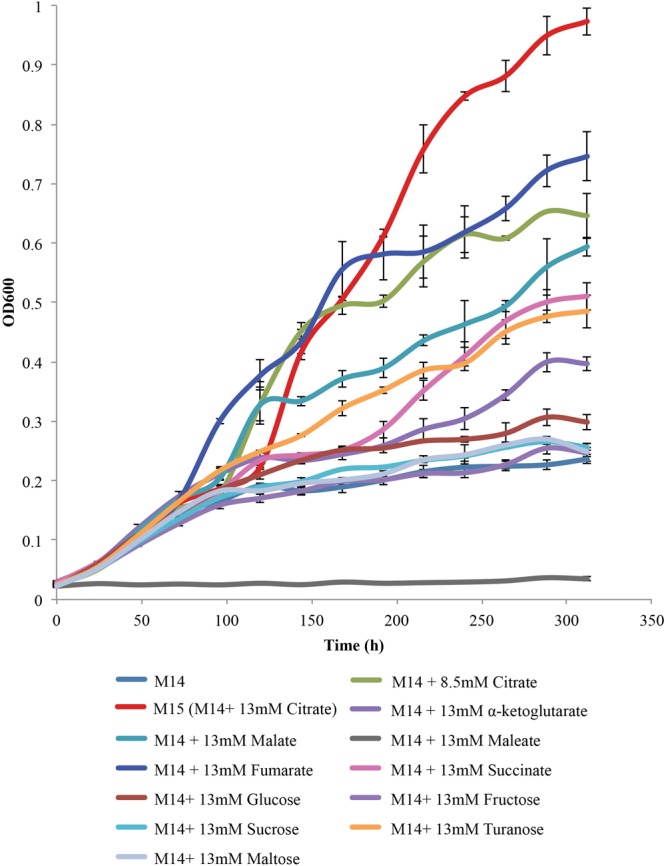
Carbohydrate source requirements for *L. crescens* growth with each of the sugars and organic acids at a concentration of 13 mM in M14 medium. A concentration of 13 mM is equivalent to 2.5 g/L citrate, the optimal level for growth of *L. crescens*. The sugars and organic acids present include α-ketoglutarate, malate, succinate, fumarate, citrate, glucose, fructose, turanose, and sucrose. M14 with 13 mM citrate is referred to as M15 medium.

The next step was to determine whether citrate was a preferred substrate for the growth of *L. crescens*. To perform this experiment, a concentration for each organic acid or sugar was chosen (13 mM) that was equal to the optimal level obtained for growth on citrate. In addition, a level of 8.5 mM citrate was used as this is the average level of citrate found in 14 citrus varieties and in the Asian citrus psyllid (Killiny, 2017; Killiny et al., 2017a). Each of these organic acids was added to the basal M14 medium as described in Step 3 above (**Figure 5**). The organic acids and sugars chosen were citrate, malate, fumarate, α-ketoglutarate, maleate, glucose, sucrose, fructose, turanose, and maltose. Although a significant lag period of over 100 h was present, 13 mM citrate provided the greatest growth for an *L. crescens* culture. The level of citrate present in phloem and the psyllid (8.5 mM) showed less growth but was still higher than any other carbohydrate source except fumarate. Growth was high on fumarate with a lower lag period than with citrate. The next best organic acid was malate followed by succinate and α-ketoglutarate. The only sugar that provided growth near that of the organic acids was turanose. Modest growth was obtained from the others. Hence, M15 is the best defined medium available for the growth of *L. crescens* to date.

## Discussion

Citrus greening disease has devastated the citrus industry in many parts of the sub-tropics, particularly in Florida. To date, there is no effective solution to combat the as-yet uncultured, causal agent, *Ca*. L. asiaticus. Without a culture, understanding the biology of this pathogen is challenging. Fortunately, a closely related cultured organism, *L. crescens*, is available and its cultures grow slowly on a very complex medium, BM-7 (Fagen et al., 2012, 2014b). With a defined medium for *L. crescens*, the growth requirements of this organism can be more precisely studied. Here, defined media were used to identify citrate as the preferred carbohydrate source for *L. crescens*. The strain can also use malate and α-ketoglutarate but neither of those organic acids provide the level of growth of a citrate-containing medium.

A defined medium was designed based on the subtraction of certain elements of the BM-7 medium, by metabolomic analysis of Grace’s insect medium from three sources, and an examination of those sugars and organic acids required for growth. The result was M15 medium which contains citrate as the sole organic acid and no sugars. As comparative genomic analyses suggest that the carbon metabolism of the *Ca*. Liberibacter pathogens is very similar to *L. crescens* including complete TCA cycles (Fagen et al., 2014a), citrate is expected to be an important carbon course for the pathogens as well.

The level of citrate that provides optimal growth for *L. crescens* is strikingly similar to the levels of citrate found in the two habitats of *Ca*. L. asiaticus, the Asian citrus psyllid and citrus phloem. The mean concentration of citrate in the phloem of 14 citrus varieties was found to be 8.39 mM (Killiny, 2017, Supplementary Table S2). Also, metabolomic analysis of the Asian Citrus Psyllid (Killiny, 2017; Killiny et al., 2017a) shows that the hemolymphs of infected and uninfected nymphs possess citrate levels of 8.24 (+1.35) mM and 2.67 (+1.09) mM, respectively. Coincidentally, the of optimal level of citrate for the culture growth of *L. crescens* discovered here was 13 mM with less growth observed at 5 and 26 mM. At 8.5 mM, citrate performs very well as a substrate for *L. crescens* (**Figure 4**). Hence the CLas habitats appear to possess a level of citrate that is nearly optimal for the growth of *Liberibacter*. Although fumarate is also a good substrate for *L. crescens* growth, it is found in very low levels in citrus phloem and was not detected in the psyllid vector (Killiny, 2017; Killiny et al., 2017a). Thus, fumarate is not expected to be a substrate for CLas growth.

As citrate appears to be such an important substrate for the growth of *Liberibacter*, an understanding of citrate uptake in these organisms is worth considering. None of the currently available genomes for *L. crescens* and the uncultured *Ca*. Liberibacter pathogens encode for known specific citrate transporters. However, these genomes do code for several transporters of unknown function. In addition, each of these genomes possesses the *dctA* gene which is well-characterized in other Rhizobiaceae (Watson et al., 1988; Yurgel et al., 2000; Yurgel and Kahn, 2004; Wang and Trivedi, 2013). DctA facilitates the uptake of C_4_ dicarboxylates in a Na^+^ or H^+^ symport dependent manner (Baker et al., 1996; Yurgel et al., 2000; Janausch et al., 2002; Yurgel and Kahn, 2004; Youn et al., 2009). However, DctA has never been shown to support citrate transport.

Given the similarity in citrate levels in the psyllid vector, citrus phloem, and in M15 medium, it appears that *Liberibacter* evolved to take advantage of citrate levels in phloem and also to manipulate the insect vector to produce more citrate (Killiny et al., 2017b). But why would *Liberibacter* evolve to use citrate as a primary carbon and energy source? Speculation on this leads to a possible control mechanism for citrus greening disease. Plants load citrate in phloem in response to phosphorous deficiency (Hoffland et al., 1992; Gaume et al., 2001; Ivanova et al., 2006; Gerke, 2015; Chen and Liao, 2016). Citrate is then transported to roots and exuded into soil where insoluble phosphate is solubilized (Hoffland et al., 1992; Gaume et al., 2001; Ivanova et al., 2006). Soluble phosphate is then taken up by the plant thereby reducing P deficiency symptoms (Shen et al., 2011; Chen and Liao, 2016). In Florida, phosphorous levels in soils are usually high but often in insoluble form. Phosphate fertilization has been low for over 60 years compared to levels of nitrogen and potassium applied (Reitz, 1958; Zekri and Obreza, 2013). As a result, citrus may load high levels of citrate into phloem to mine phosphate from soil. Those citrate levels, along with other nutrients, make citrus phloem, an ideal habitat for *Liberibacter*.

An miRNA associated with phosphorous deficiency in plants was identified in citrus tissues infected with *Ca*. L. asiaticus (Zhao et al., 2013). Zhao et al. (2013) then showed that foliar phosphate fertilization of infected citrus reduced symptom severity and improved yield compared to untreated control. The work presented here suggests that the mechanism by which foliar phosphate fertilization reduces symptoms is through a reduction in phloem citrate levels, thereby reducing the number and vigor of the pathogen’s cells in phloem. The direct link between citrate levels in phloem and the titer of the pathogen in phloem remains to be tested.

## Author Contributions

MC-M, ARC, JRP, and EWT developed the defined media and contributed to the writing. JRP and AM-B assisted MC-M in the experiments. NK performed the metabolomics presented. JCD assisted with the writing and the interpretation. EWT led the work, including the experimental design and led the writing and interpretation. All authors contributed to the writing.

## Conflict of Interest Statement

The authors declare that the research was conducted in the absence of any commercial or financial relationships that could be construed as a potential conflict of interest.
