# Supplementary material for: Development of Chemically Defined Media Reveals Citrate as Preferred Carbon Source for Liberibacter Growth
Source: Front Microbiol. 2018 Apr 5;9:668. doi: 10.3389/fmicb.2018.00668 (PMC5895721; doi:10.3389/fmicb.2018.00668)
Supplement: Supplementary file 1 [file Table_1.DOCX]

Supplemental Table 1. Chemically defined media for *L. crescens* obtained through metabolomics of Hi-GI media. Concentrations are given in mg/L.

| Components | M13 | M14 |
| --- | --- | --- |
| Inorganic salts |  |  |
| Calcium chloride dehydrate | 1320 | 1320 |
| Magnesium chloride anhydrous | 1068.2 | 1068.2 |
| Magnesium sulfate anhydrous | 1356.7 | 1356.7 |
| Potassium chloride | 2240 | 2240 |
| Sodium phosphate monobasic monohydrate | 1007 | 1007 |
| Amino acids |  |  |
| β-alanine | 447.25 | 447.25 |
| L-alanine | 447.25 | 447.25 |
| L-arginine-HCl | 1777 | 1777 |
| L-asparagine monohydrate | 1075.45 | 1075.45 |
| L-aspartic acid | 818.6 | 818.6 |
| L-cystine-2HCl | 56.38 | 56.38 |
| L-glutamic acid | 1502.2 | 1502.2 |
| L-glutamine | 358.04 | 358.04 |
| Glycine | 859.512 | 859.512 |
| L-histidine hydrochloride monohydrate | 2366.11 | 2366.11 |
| L-isoleucine | 687.36 | 687.36 |
| L-leucine | 592.89 | 592.89 |
| L-lysine-HCl | 1464.85 | 1464.85 |
| L-methionine | 678.9 | 678.9 |
| L-phenylalanine | 789.62 | 789.62 |
| L-proline | 940.61 | 940.61 |
| DL-serine | 944.76 | 944.76 |
| L-threonine | 459.8 | 459.8 |
| L-tryptophan | 373.73 | 373.73 |
| L-tyrosine disodium salt | 391.37 | 391.37 |
| L-valine | 644.31 | 644.31 |
| Betaine | 0.31 | 0.31 |
| Ornithine | 229.96 | 229.96 |
| Methionine sulfoxide | 18.2 | 18.2 |
| Vitamins |  |  |
| D-Biotin | 0.1 | 0.1 |
| Choline Chloride | 1000 | 1000 |
| Folic Acid | 0.2 | 0.2 |
| Myo-Inositol | 0.2 | 0.2 |
| Niacin | 0.2 | 0.2 |
| D-Calcium pantothenate | 0.2 | 0.2 |
| Para-aminobenzoic acid (PABA) | 0.2 | 0.2 |
| Pyrodoxine-HCl | 0.2 | 0.2 |
| Riboflavin | 0.2 | 0.2 |
| Thiamine-HCl | 0.2 | 0.2 |
| Sugars and organic acids |  |  |
| D (+) Glucose | 5667.7 | - |
| D (-) Fructose | 293.66 | - |
| Fumaric acid, free acid | 4.8 | - |
| α -ketoglutaric acid | 203.1 | - |
| L (-) Malic acid, free acid | 48.27 | - |
| Maleic acid | 4.64 | - |
| Succinic acid | 8.27 | - |
| Sucrose | 7660.6 | - |
| Turanose | 154.03 | - |
| Maltose | 616.14 | - |
| pH | 5.92 | 5.92 |
