# Supplementary material for: Development of Chemically Defined Media Reveals Citrate as Preferred Carbon Source for Liberibacter Growth
Source: Front Microbiol. 2018 Apr 5;9:668. doi: 10.3389/fmicb.2018.00668 (PMC5895721; doi:10.3389/fmicb.2018.00668)
Supplement: Supplementary file 2 [file Table_2.DOCX]

**Supplemental Table 2.** Metabolites detected in Hi-GI through quantitative metabolomics. Three Hi-GI biological replicates were derivatized using Trimethylsilyl (TMS) and Methyl Chloroformate (MCF) prior detection and quantitation through GC-MS. Concentrations of amino acids obtain through MCF and concentrations of sugars and organic acids from TMS were used as the base to elaborate the chemically defined media for *L. crescens.* Concentrations are given in mg/L. RT=Retention time. SD=Standard Deviation.

| MCF derivatization | | | |  | TMS derivatization | | |
| --- | --- | --- | --- | --- | --- | --- | --- |
| Metabolite | **RT** | **Concentration** | |  | **Metabolite** | **Concentration** | |
|  |  | **Mean** | **SD** |  |  | **Mean** | **SD** |
| Fumaric acid | 6.71 | 3.48 | 2.32 |  | Alanine | 241.43 | 193.33 |
| Maleic acid | 6.77 | 4.64 | 1.16 |  | Valine | 589.27 | 235.47 |
| Succinic acid | 6.94 | 8.27 | 2.36 |  | Phosphoric acid | 1145.55 | 240.09 |
| Glycine | 8.88 | 859.51 | 51.05 |  | Iso-Leucine | 855.25 | 153.47 |
| Alanine | 8.9 | 447.23 | 32.96 |  | Proline | 1131.73 | 206.08 |
| Valine | 10.89 | 644.33 | 43.35 |  | Glycine | 1962.99 | 367.08 |
| Leucine | 12.13 | 592.89 | 48.53 |  | Serine | 1316.78 | 230.15 |
| Iso-leucine | 12.31 | 687.35 | 76.08 |  | Threonine | 4824.33 | 2076.25 |
| Threonine | 12.38 | 459.8 | 95.3 |  | β-Alanine | 278.86 | 63.26 |
| Malic acid | 12.58 | 0 | 0 |  | Malic acid | 48.27 | 60.34 |
| Proline | 12.77 | 940.61 | 69.08 |  | Aspartic acid | 1299.15 | 274.21 |
| Asparagine | 12.74 | 1075.44 | 223.28 |  | Pyroglutamic acid | 1114.25 | 173.01 |
| Quinic acid | 13.3 | 0 | 0 |  | 2-Ketoglutaric acid | 203.09 | 67.21 |
| Aspartic acid | 13.68 | 818.63 | 331.44 |  | Glutamic acid | 1047.57 | 303.09 |
| Citric acid | 13.72 | 0 | 0 |  | Phenylalanine | 607.9 | 173.45 |
| Serine | 14.56 | 944.76 | 244.86 |  | Asparagine | 260.27 | 342.19 |
| Glutamine | 14.56 | 358.04 | 115.45 |  | Ornithine | 229.96 | 179.74 |
| Glutamic acid | 15.22 | 1502.2 | 406.08 |  | Tyrosine | 11161.3 | 2815.69 |
| Methionine | 15.4 | 678.91 | 83.56 |  | Fructose | 293.66 | 100.89 |
| Cysteine | 16.45 | 21.81 | 3.63 |  | Glucose | 5667.7 | 288.25 |
| Phenylalanine | 16.8 | 789.61 | 79.29 |  | Sucrose | 7660.6 | 5757.43 |
| Lysine | 19.8 | 1172.44 | 192.97 |  | Turanose | 154.04 | 37.65 |
| Ferulic acid | 20.35 | 0 | 0 |  | Maltose | 616.14 | 112.96 |
| Histidine | 20.44 | 2366.11 | 467.02 |  |  |  |  |
| Tyrosine | 21.43 | 391.37 | 50.73 |  |  |  |  |
| Tryptophan | 23.14 | 373.73 | 110.28 |  |  |  |  |
