# Supplementary material for: Development of Chemically Defined Media Reveals Citrate as Preferred Carbon Source for Liberibacter Growth
Source: Front Microbiol. 2018 Apr 5;9:668. doi: 10.3389/fmicb.2018.00668 (PMC5895721; doi:10.3389/fmicb.2018.00668)
Supplement: Supplementary file 3 [file Table_3.docx]

**Supplemental Table 3.** Metabolites detected in Hi-GI through quantitative metabolomics. Three biological replicates from each media were derivatized using Methyl Chloroformate (MCF) prior detection and quantitation through GC-MS. Concentrations of amino acids obtain through MCF were used as the base to elaborate the chemically defined media for *L. crescens.* Concentrations are given in mM. RT=Retention time. SD=Standard Deviation. tr=trace (under the limit of quantification)

|  |  | **Hi-G** | | **Lab-GI** | | **GI-GI** | |
| --- | --- | --- | --- | --- | --- | --- | --- |
| **Compound** | **RT** | **average** | **STD** | **average** | **STD** | **average** | **STD** |
| Fumaric | 6.71 | 0.03 (tr) | 0.02 | 0.37 | 0.06 | 0.41 | 0.04 |
| Maleic | 6.77 | 0.04 (tr) | 0.01 | 0.43 | 0.08 | 0.40 | 0.05 |
| Succinic acid | 6.94 | 0.07 (tr) | 0.02 | 0.56 | 0.03 | 0.51 | 0.05 |
| Glycine | 8.88 | 11.45 | 0.68 | 8.38 | 0.55 | 7.44 | 0.81 |
| Alanine | 8.9 | 5.02 | 0.37 | 3.75 | 0.28 | 3.33 | 0.37 |
| Valine | 10.89 | 5.50 | 0.37 | 1.90 | 0.15 | 1.54 | 0.36 |
| Leucine | 12.13 | 4.52 | 0.37 | 1.25 | 0.10 | 1.06 | 0.14 |
| Iso-leucine | 12.31 | 5.24 | 0.58 | 0.92 | 0.10 | 0.75 | 0.10 |
| Threonine | 12.38 | 3.86 | 0.80 | 1.38 | 0.18 | 0.92 | 0.27 |
| Malic acid | 12.58 | 0.00 | 0.00 | 3.66 | 0.38 | 3.09 | 0.35 |
| Proline | 12.77 | 8.17 | 0.60 | 4.86 | 2.39 | 5.50 | 0.53 |
| Asparagine | 12.74 | 8.14 | 1.69 | 2.88 | 0.36 | 2.48 | 0.38 |
| Quinic acid | 13.3 | 0.00 | 0.00 | 0.00 | 0.00 | 0.02 | 0.03 (tr) |
| Aspartic acid | 13.68 | 6.15 | 2.49 | 4.20 | 0.25 | 3.69 | 0.64 |
| Citric acid | 13.72 | 0.00 | 0.00 | 0.00 | 0.00 | 0.02 | 0.02 (tr) |
| Serine | 14.56 | 8.99 | 2.33 | 11.12 | 3.47 | 4.45 | 1.06 |
| Glutamine | 14.56 | 2.45 | 0.79 | 1.14 | 0.22 | 0.98 | 0.19 |
| Glutamic acid | 15.22 | 10.21 | 2.76 | 5.37 | 0.70 | 4.43 | 0.77 |
| Methionine | 15.4 | 4.55 | 0.56 | 0.43 | 0.06 | 0.35 | 0.05 |
| Cystein | 16.45 | 0.18 | 0.03 | 0.00 | 0.00 | 0.00 | 0.01 (tr) |
| Phenylalanine | 16.8 | 4.78 | 0.48 | 1.85 | 0.17 | 1.61 | 0.15 |
| Lysine | 19.8 | 8.02 | 1.32 | 4.27 | 0.78 | 4.90 | 0.71 |
| Ferulic acid | 20.35 | 0.00 | 0.00 | 0.00 | 0.00 | 0.00 | 0.00 |
| Histidine | 20.44 | 15.25 | 3.01 | 13.40 | 1.96 | 9.56 | 3.34 |
| Tyrosine | 21.43 | 2.16 | 0.28 | 0.70 | 0.16 | 0.42 | 0.08 |
| Tryptophan | 23.14 | 1.83 | 0.54 | 1.21 | 0.24 | 1.01 | 0.18 |
