# Supplementary material for: Development of Chemically Defined Media Reveals Citrate as Preferred Carbon Source for Liberibacter Growth
Source: Front Microbiol. 2018 Apr 5;9:668. doi: 10.3389/fmicb.2018.00668 (PMC5895721; doi:10.3389/fmicb.2018.00668)
Supplement: Supplementary file 4 [file Table_4.docx]

**Supplemental Table 4.** Metabolites detected in Hi-GI through quantitative metabolomics. Three biological replicates from each media were derivatized using Trimethylsilyl (TMS) prior detection and quantitation through GC-MS. Concentrations concentrations of sugars and organic acids from TMS were used as the base to elaborate the chemically defined media for *L. crescens.* Concentrations are given in mM. SD=Standard Deviation.

|  | **Gibco  Media** | | **Hi  Media** | | **Lab Media** | |
| --- | --- | --- | --- | --- | --- | --- |
| **TMS metabolite** | **Mean** | **SD** | **Mean** | **SD** | **Mean** | **SD** |
| Alanine | 1.22 | 0.90 | 2.71 | 2.17 | 1.22 | 0.48 |
| Valine | 0.68 | 0.47 | 5.03 | 2.01 | 0.73 | 0.32 |
| Phosphoric acid | 2.82 | 0.53 | 11.69 | 2.45 | 3.33 | 0.22 |
| Iso-Leucine | 0.42 | 0.15 | 6.52 | 1.17 | 0.45 | 0.14 |
| Proline | 2.47 | 1.40 | 9.83 | 1.79 | 2.12 | 1.08 |
| Glycine | 14.73 | 3.25 | 26.15 | 4.89 | 16.20 | 0.85 |
| Serine | 4.72 | 1.30 | 12.53 | 2.19 | 10.74 | 2.20 |
| Threonine | 10.27 | 2.21 | 40.50 | 17.43 | 16.75 | 11.01 |
| β-Alanine | 2.93 | 0.96 | 3.13 | 0.71 | 3.26 | 0.73 |
| Malic acid | 5.71 | 1.03 | 0.36 | 0.45 | 6.31 | 1.14 |
| Aspartic acid | 0.53 | 0.15 | 9.76 | 2.06 | 0.84 | 0.38 |
| Pyroglutamic acid | 0.93 | 0.11 | 8.63 | 1.34 | 0.89 | 0.60 |
| PGA derivative | 0.89 | 0.41 | 1.65 | 0.55 | 1.02 | 0.38 |
| 2-Ketoglutaric acid | 7.11 | 2.13 | 1.39 | 0.46 | 8.23 | 3.39 |
| Glutamic acid | 0.25 | 0.09 | 7.12 | 2.06 | 0.39 | 0.33 |
| Phenylalanine | 0.22 | 0.02 | 3.68 | 1.05 | 0.23 | 0.08 |
| Asparagine | 0.41 | 0.07 | 1.97 | 2.59 | 0.44 | 0.06 |
| Sugar Furanose trace | 0.21 | 0.16 | 0.88 | 0.86 | 0.08 | 0.06 |
| Ornithine | 0.36 | 0.06 | 1.74 | 1.36 | 0.34 | 0.03 |
| Tyrosine | 5.73 | 1.82 | 61.60 | 15.54 | 3.84 | 2.04 |
| Fructose | 6.67 | 1.10 | 1.63 | 0.56 | 7.58 | 0.86 |
| Glucose | 9.76 | 1.53 | 31.46 | 1.60 | 11.75 | 0.94 |
| Unk disaccharide trace | 0.41 | 0.29 | 0.43 | 0.20 | 0.50 | 0.22 |
| Unk *m/z* 456/513 | 0.28 | 0.02 | 0.32 | 0.07 | 0.25 | 0.02 |
| Unk disaccharide *m/z* 361 | 0.34 | 0.15 | 0.43 | 0.17 | 0.34 | 0.10 |
| Sucrose | 19.79 | 14.60 | 22.38 | 16.82 | 32.27 | 16.56 |
| Turanose | 0.34 | 0.17 | 0.45 | 0.11 | 0.42 | 0.06 |
| Maltose | 0.23 | 0.09 | 1.80 | 0.33 | 0.16 | 0.03 |
| Unk disaccharide *m/z* 361 | 0.72 | 0.37 | 0.76 | 0.11 | 0.95 | 0.12 |
